# Using previously registered cone beam computerized tomography images to facilitate online computerized tomography to cone beam computerized tomography image registration in lung stereotactic body radiation therapy

**DOI:** 10.1002/acm2.13549

**Published:** 2022-02-02

**Authors:** Jian Liang, Qiang Liu, Inga Grills, Thomas Guerrero, Craig Stevens, Di Yan

**Affiliations:** ^1^ Beaumont Health System, Royal Oak Radiation Oncology Michigan USA

**Keywords:** CBCT, IGRT, image registration, lung SBRT

## Abstract

**Purpose:**

In our conventional image registration workflow, the four‐dimensional (4D) CBCT was directly registered to the reference helical CT (HCT) using a dual registration approach within the Elekta XVI software. In this study, we proposed a new HCT–CBCT auto‐registration strategy using a previously registered CBCT (CBCTpre) as the reference image and tested its clinical feasibility.

**Methods:**

From a previous CBCT session, the registered average 4D CBCT was selected as CBCTpre and the HCT–CBCTpre registration vector from the clinician's manual registration result was recorded. In the new CBCT session, auto‐registration was performed between the new average 4D CBCT (CBCTtx) and CBCTpre (CBCTpre‐CBCTtx). The overall HCT–CBCTtx registration result was then derived by combing the results from two registrations (i.e., HCT–CBCTpre + CBCTpre–CBCTtx). The results from the proposed method were compared with clinician's manually adjusted HCT–CBCTtx registration results (“ground truth”) to evaluate its accuracy using a test dataset consisting of 32 challenging registration cases.

**Results:**

The uncertainty of the proposed auto‐registration method was −0.1 ± 0.5, 0.1 ± 1.0, and −0.1 ± 0.7 mm in three translational directions (lateral, longitudinal, and vertical) and 0.0° ± 0.9°, 0.3° ± 0.9°, and 0.4° ± 0.7° in three rotation directions, respectively. Two patients (6.3%) had translational uncertainty > 2 mm (max = 3.1 mm) and both occurred in the longitudinal direction. Meanwhile, the uncertainty of the conventional direct HCT–CBCTtx auto‐registration was −0.4 ± 2.6, −0.2 ± 7.4, −1.4 ± 3.6 mm for translations and −0.3° ± 1.2°, 0.0° ± 1.6°, and 0.1 ± 1.1° for rotations. Eleven patients (34.4%) had translation uncertainty > 2 mm (max = 26.2 mm) in at least one direction. Accuracy in translation was improved with the new method, while rotation accuracy stayed in the same order.

**Conclusion:**

We demonstrated the feasibility of incorporating prior clinical registration knowledge into the online HCT–CBCT registration process. The proposed auto‐registration method provides a quick and reliable starting solution for online HCT–CBCT registration.

## 1 INTRODUCTION

Stereotactic body radiation therapy (SBRT) has been established as an effective and safe treatment option for patients with small primary non‐small cell lung cancers (NSCLCs) or lung metastases.^1^, ^2^ Image guidance is crucial in lung SBRT delivery given the day‐to‐day internal change of tumor position and the small treatment margins employed to reduce RT‐related toxicities.^3^, ^4^, ^5^ Among various image guidance modalities, the linear accelerator gantry‐mounted kilovoltage (kV) cone beam computerized tomography (CBCT) has become one of the most frequently used imaging technologies.^6^ The Elekta X‐ray Volume Imaging (XVI) four‐dimensional (4D) CBCT system (Symmetry™, Elekta Oncology Systems Ltd, Crawley, UK) has been incorporated into our lung SBRT workflow. A typical 4D Symmetry™ protocol acquires approximately 1320 projection images over a 200° gantry rotation with a total acquisition time of ∼4 min. Reconstruction produces 10 phase‐based 4D CBCT images and one average 4D CBCT image, which are subsequently registered to the reference helical CT (HCT) using a dual registration method available in the XVI software. The dual registration is a two‐step registration procedure with each step focusing on different regions of interest (ROIs) and using different characteristics of the image content, including: (1) a bone registration within a large ROI to ensure proper overall patient alignment (i.e., clip‐box registration), and (2) a soft tissue registration within a small ROI focusing on tumor match (i.e., mask registration).

Automatic image registration between HCT and CBCT may be challenging due to the degraded image quality of CBCT caused by increased scattered radiation and various artifacts inherent to CBCT. In most of the clinical situations where the tumor boundaries are well‐defined, the XVI automatic registration process performs well with minor user intervention. However, large manual adjustment often becomes necessary in certain challenging cases, such as tumors with low visibility, large degree of motions, and radiographic changes as a response to the radiation treatment. In real clinical situations, carefully performed manual registrations by experienced clinicians are generally regarded as the best estimate of the “ground truth.”^7^ However, manual registration also has its limitations, such as being time‐consuming, user‐dependent, and prone to errors especially when performed in a time‐compressed online setting.^8^, ^9^ In this paper, we propose an automatic HCT‐CBCT image registration strategy which incorporates previous registration knowledge into the online registration workflow. This new method uses previously registered CBCT (denoted as CBCTpre) as a bridge to facilitate the registration between the reference HCT and the CBCT from the current treatment session (denoted as CBCTtx). For notational simplicity, we denote it as “*HCT‐CBCTpre‐CBCTtx auto‐registration*” in the rest of this paper. In this study, we implemented the proposed method within XVI platform using the XVI registration algorithm and proved its clinical feasibility. We also compared its accuracy and robustness with the conventional “*direct HCT‐CBCTtx auto‐registration*” method.

## 2 METHODS AND MATERIALS

### 2.1 Patient selection and conventional registration method

Among ∼100 lung SBRT patients recently treated in our institution, 32 patients were selected for this institutional review board (IRB) approved the retrospective study. Direct HCT–CBCTtx image registrations of these patients were considered as challenging due to various reasons including: tumors with low‐contrast and ill‐defined tumor boundaries, large or altered tumor motions, and local radiographic changes of tumor and surrounding tissue as responses to the radiation treatment. Patient and tumor target characteristics are summarized in Table 1. All patients were immobilized using Alpha Cradle and BodyFIX vacuum system (Elekta Oncology System, Crawley, UK). 4D HCTs were acquired under free breathing condition on a 16‐slice CT scanner (Big bore, Philips Medical Systems, Cleveland, OH, USA). The average HCT image, which had a 0.98 × 0.98 mm^2^ in‐plane resolution and 3 mm slice thickness, was used for the treatment planning and also set as the reference image in XVI for image guidance. Gross tumor volumes (GTVs) from all 10 phase HCT images were combined together to form the internal target volume (ITV). Five millimeter transversal and 5–7 mm cranio‐caudal margins were added to ITV to define the planning target volume (PTV). In addition to the HCT simulation, all patients also underwent a verification simulation (i.e., a “dry run”) on the treatment machine the day before their first fraction of treatment. This verification session duplicates the steps of patient setup, imaging, and treatment without actually delivering the radiation, which allows staff time to verify the isocenter placement, check geometric clearance and troubleshoot potential problems in an organized team approach.^10^ All patients were treated with a 4–5 fractions schedule that delivered over 1–2 weeks.

**TABLE 1:** Patient and tumor target characteristics

					**Motion range on planning CT (cm)**				
**Patient #**	**Age**	**Gender**	**Target location**	**Size (GTV, cm^3^)**	**Lat**	**Ver**	**Long**	**3D**	**Target visibility on CBCT**
1	64	F	RLL	5.51	0.09	0.24	0.69	0.74	Intermediate
2	67	F	RML	8.78	0.17	0.24	0.60	0.67	Intermediate
3	58	F	RLL	3.75	0.14	0.31	0.91	0.97	Intermediate
4	66	F	LUL	1.65	0.35	0.40	0.32	0.62	Poor
5	33	F	RLL	1.81	0.07	0.24	0.74	0.79	Poor
6	78	F	RLL	3.27	0.14	0.44	0.92	1.03	Intermediate
7	81	F	RML	17.52	0.15	0.39	0.91	1.00	Intermediate
8	80	M	LUL	1.45	0.08	0.09	0.05	0.13	Intermediate
9	85	F	RLL	21.6	0.23	0.30	0.91	0.98	Intermediate
10	74	M	RLL	2.67	0.13	0.42	1.41	1.47	Intermediate
11	64	M	RML	14.96	0.39	0.82	0.32	0.96	Intermediate
12	72	M	LUL	8.99	0.07	0.19	0.10	0.23	Poor
13	58	M	RLL	21.7	0.06	0.15	0.68	0.70	Intermediate
14	77	F	LLL	3.27	0.07	0.56	0.70	0.90	Poor
15	85	M	LUL	12.7	0.21	0.40	1.78	1.84	Poor
16	63	F	RML	3.75	0.33	0.37	0.28	0.57	Intermediate
17	75	M	RLL	5.25	0.11	0.43	0.69	0.82	Intermediate
18	86	M	RLL	2.57	0.48	1.57	2.05	2.63	Intermediate
19	72	F	LUL	5.49	0.13	0.38	0.74	0.84	Poor
20	78	F	RML	1.94	0.42	0.38	1.51	1.62	Poor
21	77	M	RUL	7.23	0.10	0.27	0.24	0.38	Poor
22	80	F	LUL	2.82	0.09	0.28	0.79	0.84	Intermediate
23	83	M	LLL	1.26	0.08	0.34	0.76	0.84	Intermediate
24	81	F	LUL	9.12	0.10	0.19	0.15	0.26	Poor
25	77	M	LLL	5.16	0.18	0.18	0.62	0.67	Poor
26	64	F	RLL	15.90	0.09	0.18	0.98	1.00	Intermediate
27	78	F	RLL	16.31	0.15	0.13	1.25	1.27	Intermediate
28	78	M	LUL	3.92	0.10	0.24	0.45	0.52	Poor
29	64	F	RUL	1.9	0.04	0.21	0.30	0.37	Poor
30	66	M	RLL	17.8	0.08	0.31	1.11	1.16	Intermediate
31	60	F	RUL	10.06	0.05	0.17	0.37	0.41	Poor
32	66	F	LLL	2.64	0.28	0.33	1.51	1.57	Intermediate

*Notes*: GTV, gross tumor volume; Lat, patient left‐right direction;Long, superior‐inferior; 3D, three‐dimentional; Ver, anterior‐posterior direction

4D CBCT images were acquired for image guidance during the verification session and prior to each treatment delivery using the Elekta XVI Symmetry™ protocol. The reconstructed CBCT images (average + 10 phases) had an isotropic image resolution of 2 × 2 × 2 mm^3^. In our conventional registration workflow, the reference HCT was directly registered to the CBCT using the Elekta dual registration steps within XVI software. In the first step, “Bone (T+R)” registration was performed between the reference HCT and average CBCT using a large rectangular ROI that encompassed the PTV and nearby bony anatomy (usually vertebrae). The “Bone (T+R)” registration mode used a chamfer matching algorithm based on regions with bone densities and “(T+R)” indicated that both translational and rotational shifts were calculated in this step.^11^ In the second step, soft tissue registration was performed within a smaller mask ROI, where the mask was defined as ITV plus 5 mm margin. In this step, the “Grey Value (*T*)” registration mode was selected, where the registration algorithm used a grey level “correlation ratio” as the similarity measure and “(*T*)” indicated that only translational shift was calculated (i.e., without changing rotation angle from the previous step).^11^, ^12^, ^13^, ^14^ After these two auto‐registration steps, the auto‐result was evaluated and adjusted if necessary by the clinician by: (1) visually inspecting the tumor matching between the average HCT and the average CBCT in all three orthogonal views using a lung window/level setting; and (2) displaying a cine loop of all CBCT phase images to ensure the tumor motion was contained within the ITV.

### 2.2 Proposed HCT–CBCTpre–CBCTtx auto‐registration method

**FIGURE 1:**
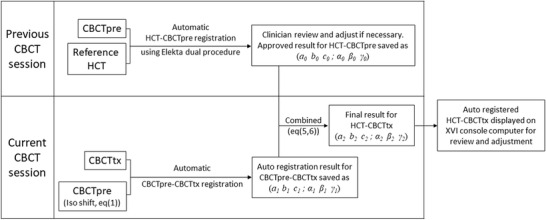
Flow chart of the proposed HCT–CBCTpre–CBCTtx auto‐registration process

As described in the introduction section, the proposed method uses previously registered CBCT (CBCTpre) as the reference image for the CBCTtx registration. Figure 1 outlines the schematic flow chart of the proposed method. Detailed implementations of each step are described below.

### 2.3 Set CBCTpre as the reference image in XVI

When patient is treated in head first supine position, in the XVI coordinate system,^11^ the *x*, *y*, and *z* coordinates are in the right–left, inferior–superior, and posterior–anterior direction, respectively. Denote $\left(a_{0},b_{0},c_{0}\right)$ as translation vector by moving the reference HCT to CBCT in the *x*, *y*, and *z*‐direction, while $\left(\alpha_{0},\beta_{0},\gamma_{0}\right)$ as rotation vector in the −*x*, +*y*, −*z* direction, respectively, the previous HCT–CBCTpre registration is expressed as:

(1) $$X_{\mathrm{pre}}-X_{\mathrm{pre}\_\mathrm{iso}}=R_{0}\left(X_{\mathrm{HCT}}-X_{\mathrm{HCT}\_\mathrm{iso}}\right)+T_{0},$$

where $X_{\mathrm{pre}}$ and $X_{\mathrm{HCT}}$ are point vectors that describe the (*x*, *y*, *z*) coordinates of each voxel within the CBCTpre and the reference HCT images, $X_{\mathrm{pre}\_\mathrm{iso}}\;$is the kV imaging center on the CBCTpre and $X_{\mathrm{HCT}\_\mathrm{iso}}\;$is the beam isocenter on the HCT image, respectively. $R_{0}=R_{z}\;\left(-\gamma_{0}\right)R_{y}\left(\beta_{0}\right)R_{x}\left(-\alpha_{0}\right)$, $T_{0}={\left\{a_{0},b_{0},c_{0}\right\}}^{T}\;$, and $R_{z},R_{y},\;$and $R_{x}$ are 3×3 matrix defined as:

(2) $$\begin{array}{ccc} & & R_{x}(t)\;=\left[\begin{array}{ccc} 1 & 0 & 0\\ 0 & \cos(t) & -\sin(t)\\ 0 & \sin(t) & \cos(t) \end{array}\right]\;,\;\;\\ & & R_{y}(t)\;=\left[\begin{array}{ccc} \cos(t) & 0 & \sin(t)\\ 0 & 1 & 0\\ -\sin(t) & 0 & \cos(t) \end{array}\right],\;\\ & & R_{z}(t)\;=\left[\begin{array}{ccc} \cos(t) & -\sin(t) & 0\\ \sin(t) & \cos(t) & 0\\ 0 & 0 & 1 \end{array}\right] \end{array}$$

The CBCTpre was first exported to our treatment planning system (Pinnacle 16.2, Philips Radiation Oncology Systems, Fitchburg, WI) in Digital Imaging and Communications in Medicine (DICOM) format. In Pinnacle, a new plan dataset was created using the CBCTpre as the reference image and the planning HCT as the secondary image. Image fusion between CBCTpre and HCT in the Pinnacle format based on Equation (1), and ROIs on the HCT were subsequently transferred to the CBCTpre. An artificial beam was then created with beam isocenter defined by

(3) $$\mathrm{Beam}\;\mathrm{Iso}\;\mathrm{At}\;\mathrm{CBCTpre}\;=\;X_{\mathrm{pre}\_\mathrm{iso}}+\;T_{0}.$$

It is necessary to note here that small image shift often occurs when DICOM data are transferred between different software platforms due to the inconsistency in interpreting the DICOM data element across different vendors.^15^ In this study, it was found that a ½ pixel offset was introduced when the Elekta CBCT DICOM data were transferred from XVI to Pinnacle. This phenomenon was also observed when transferred CBCT from XVI to other imaging Picture Archiving and Communication System (PACS) systems such as Mosaiq (Elekta Oncology Systems) and MIM (MIM software Inc.). Therefore, a 1 mm correction (i.e., $\;\;X_{\mathrm{pre}\_\mathrm{iso}}=\;{\left\{0.1,-0.1,-0.1\right\}}^{T}\mathrm{cm}$) was applied to Equation (3) to account for the ½ pixel image offset. By exporting a complete reference dataset from Pinnacle back to XVI (image + beam isocenter + ROIs), a new reference treatment in XVI was created using the CBCTpre as the reference image. Hence, we could perform CBCTpre–CBCTtx auto‐registration within XVI platform using the same registration algorithm. In the XVI reference data setting, the same clip‐box and mask ROIs as those used in the conventional registration method were also created.

### 2.4 CBCTpre–CBCTtx auto‐registration

Automatic CBCTpre–CBCTtx image registration was performed in the XVI software using the same dual steps and algorithms as those used in the conventional direct HCT–CBCTtx auto‐registration. Denote $\left(a_{1},b_{1},c_{1}\right)$ and $\left(\alpha_{1},\beta_{1},\gamma_{1}\right)$ as the CBCTpre–CBCTtx auto registration outputs, we have

(4) $$X_{tx}=R_{1}\;\;\left(X_{\mathrm{pre}}-\mathrm{Beam}\;\mathrm{Iso}\;\mathrm{At}\;\mathrm{CBCTpre}\;\right)\;+\;T_{1,}\;$$

where $X_{tx}\;$denotes a point vector on the CBCTtx image, $R_{1}=R_{z}\;\left(-\gamma_{1}\right)R_{y}\left(\beta_{1}\right)R_{x}\left(-\alpha_{1}\right)$ and $T_{1}={\left\{a_{1},b_{1},c_{1}\right\}}^{T}\;$, respectively. It is worth to point out that the $X_{tx}$ in Equation (4) uses the coordinate defined within the XVI software, where the kV imaging center is set at ${\left\{0,\;0,\;0\right\}}^{T}$. Hence, the variable for the kV imaging center of CBCTtx is dropped in Equation (4) for simplicity.

### 2.5 Combine two registrations to obtain final HCT–CBCTtx registration result

Merging Equations (1) and (3) into Equation (4) yields

(5) $$X_{tx}=R_{1}\;R_{0}\left(X_{\mathrm{HCT}}-X_{\mathrm{HCT}\_\mathrm{iso}}\right)+T_{1}.$$

From Equation (5), we can find that the final translation vector $\left(a_{2},b_{2},c_{2}\right)$ of the HCT–CBCTtx registration is simply the vector $T_{1}$, while the rotation matrix is $R_{1}R_{0}$ and the three final rotation parameters $(\alpha_{2},\beta_{2},\gamma_{2})\;$can be subsequently solved by

(6) $$R_{z}\left(-\gamma_{2}\right)R_{y}\left(\beta_{2}\right)R_{x}\;\left(-\alpha_{2}\right)=\;R_{1}\;R_{0.}$$

It is necessary to point out that, although not explicitly expressed here, the translation vector $T_{0}$ from the previous HCT–CBCTpre registration is already considered in Equation (3) when creating the new reference dataset using CBCTpre.

### 2.6 Validation and data analysis

For each patient, CBCT images from the verification session and the last treatment session were selected as the CBCTpre and CBCTtx, respectively. The clinical HCT–CBCTpre registration result stored in XVI database was taken as the prior registration knowledge (i.e., $\left(a_{0},b_{0},c_{0}\right)$ and $\left(\alpha_{0},\beta_{0},\gamma_{0}\right)$). For the CBCTtx, the conventional HCT–CBCTtx registration procedure as described in Section 2.1 was retrospectively repeated by three independent users sufficiently skilled with the XVI software. The average of three users’ result was taken as the manual consensus “ground truth” and used to evaluate the accuracy of the proposed method. It is worth to mention that the registration results from three users showed relatively small inter‐observer variation with standard deviations of 0.2, 0.3, and 0.3 mm for three translational and 0.7, 0.8, and 0.5 degree for three rotational parameters, respectively. In comparison, larger inter‐observer variations, at the order of 1–2 mm, had been reported by other studies for CBCT‐based image guidance in lung SBRT.^8^, ^16^

As described in Section 2.2.2, the CBCTpre–CBCTtx registration step was carried out in XVI in an automatic manner, that is, without any human interference or adjustment. The result from this step (i.e., $\left(a_{1},b_{1},c_{1}\right)$ and $\left(\alpha_{1},\beta_{1},\gamma_{1}\right)$) was then combined with the HCT–CBCTpre result (i.e., $\left(a_{0},b_{0},c_{0}\right)$and $\left(\alpha_{0},\beta_{0},\gamma_{0}\right)$) to calculate the six final parameters for the HCT–CBCTtx registration (i.e., $\left(a_{2},b_{2},c_{2}\right)$ and $\left(\alpha_{2},\beta_{2},\gamma_{2}\right)$). Residual error of each final parameter was calculated to quantify the accuracy of the proposed method, where each residual error was defined as the difference between the HCT–CBCTpre‐CBCTtx auto‐result and its corresponding manual “ground truth” value. For translational shifts, a 3D residual vector was also calculated to depict the amount of overall displacement in three‐dimensions. Results of direct HCT–CBCTtx auto‐registration, that is, prior to making any manual adjustment, were also collected and analyzed. Residual errors from two auto‐registration methods were compared to assess the potential improvement by the proposed method. To test the statistical significance of the difference between the group means, Student paired *t*‐test was performed if the Shapiro–Wilk normality test passed (*p* > 0.05), otherwise the Wilcoxon signed rank test was used. The results were considered significant for *p* < 0.05.

## 3 RESULTS

Figure 2a shows registration errors in three translational directions for individual patients. Data of the patient group are also summarized using box‐and‐whisker plots and displayed on the right side of the same figure. Registration errors from the proposed HCT–CBCTpre–CBCTtx method, represented by red colors, averaged at −0.1±0.5, 0.1±1.0, and −0.1±0.7 mm in *Tx* (lateral), *Ty* (longitudinal), and *Tz* (vertical), respectively. Most of the data points fell within the ±2 mm bound lines, which represented the upper range of the typical inter‐observer variation of manual image registration in lung SBRT.^8^, ^16^, ^17^ Two patients (6.3%) had translational uncertainty > 2 mm (max = 3.1 mm), and both occurred in the longitudinal direction. Meanwhile, corresponding errors from the direct HCT–CBCTtx auto‐registration, represented by blue colors, were −0.4±2.6, −0.2±7.4, −1.4±3.6 mm, respectively, with 11 patients (34.4%) had translation error > 2 mm (max = 26.2 mm) in at least one direction. The proposed method exhibited smaller mean for the patient group compared to direct HCT–CBCTtx auto‐registration, however, statistical analysis showed that the differences did not reach the statistical significance level. Figure 2b shows the overall errors in three dimensions. Twelve patients (37.5%) had > 2 mm 3D errors from the direct HCT–CBCTtx auto‐registration, while the number decreased to four patients for the proposed method. The improved consistency was illustrated by the smaller interquartile range and the lack of outliers in the box‐and‐whisker plot. For 3D errors, statistical analysis showed that the difference in group mean reached the statistical significance level (*p* = 0.0075).

FIGURE 2(a) Translation registration errors in *Tx* (lateral), *Ty* (longitudinal), and *Tz* (vertical) direction, and (b) in three‐dimensional (3D) for individual patient and box‐and‐whisker plot for patient group. The solid dot and the line in the box represent the mean and median, respectively. The upper and lower edges of the box indicate the interquartile range (i.e., the range of values between the 25th and 75th percentiles). The upper and lower whiskers lines indicate the range of data within 1.5 × interquartile range from the upper and lower edges of the box, respectively, and the cross sign in the plots indicates the outliers beyond the whiskers. The *p*‐values for comparisons of group mean between two auto‐registration methods are also stated

**Figure:**
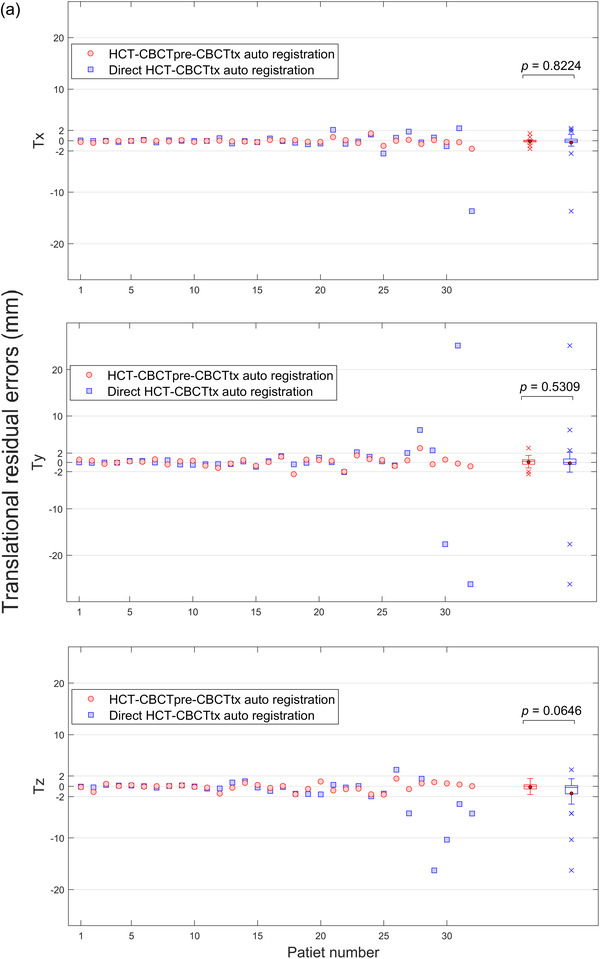

**Figure:**
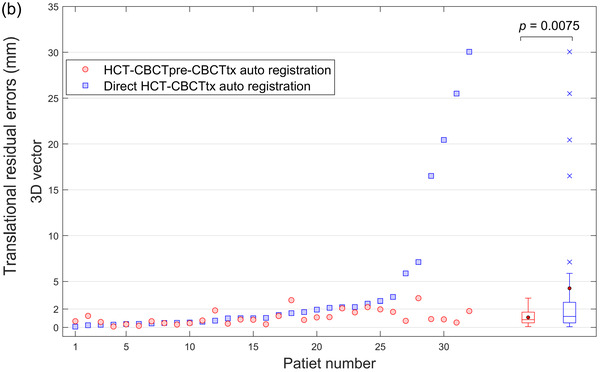

**FIGURE 3:**
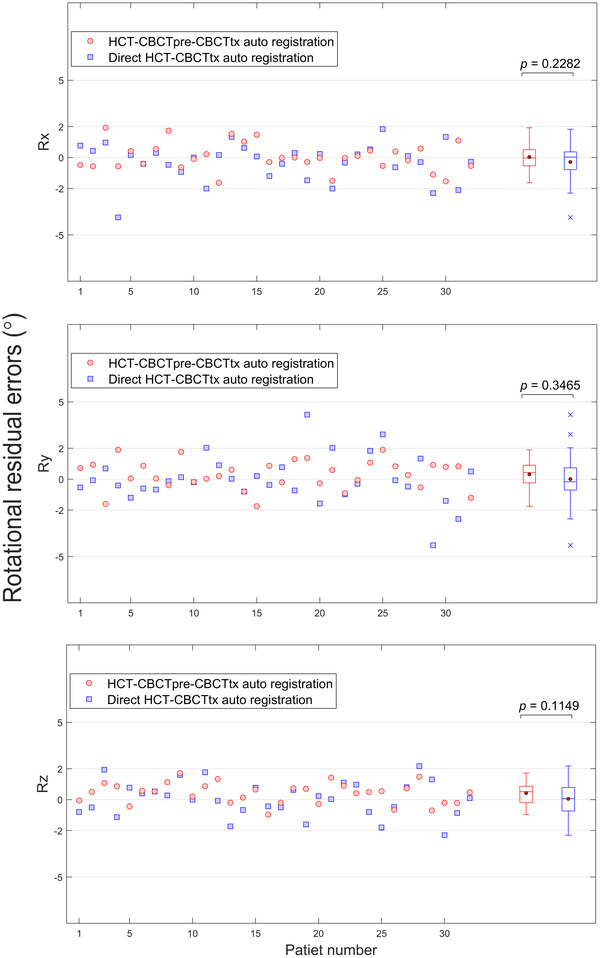
Rotation registration errors about the *x* (lateral), *y* (longitudinal), *z* (vertical) axis for individual patient and box‐and‐whisker plot for patient group. The solid dot and the line in the box represent the mean and median, respectively. The upper and lower edges of the box indicate the interquartile range (i.e., the range of values between the 25th and 75th percentiles). The upper and lower whiskers lines indicate the range of data within 1.5 × interquartile range from the upper and lower edges of the box, respectively, and the cross sign in the plots indicates the outliers beyond the whiskers. The *p* values for comparisons of group mean between two auto‐registration methods are also stated

Figure 3 summarizes the residual errors of three rotational parameters. Compared with the residual errors from direct HCT–CBCTtx auto‐registration which averaged at −0.3°±1.2° in *Rx*, 0.0°±1.6° in *Ry*, and 0.1°±1.1° in *Rz*, respectively, the proposed method produced comparable errors which averaged at 0.0°±0.9°, 0.3°±0.9°, and 0.4°±0.7°, respectively. These small differences were not statistically significant between two methods.

**FIGURE 4:**
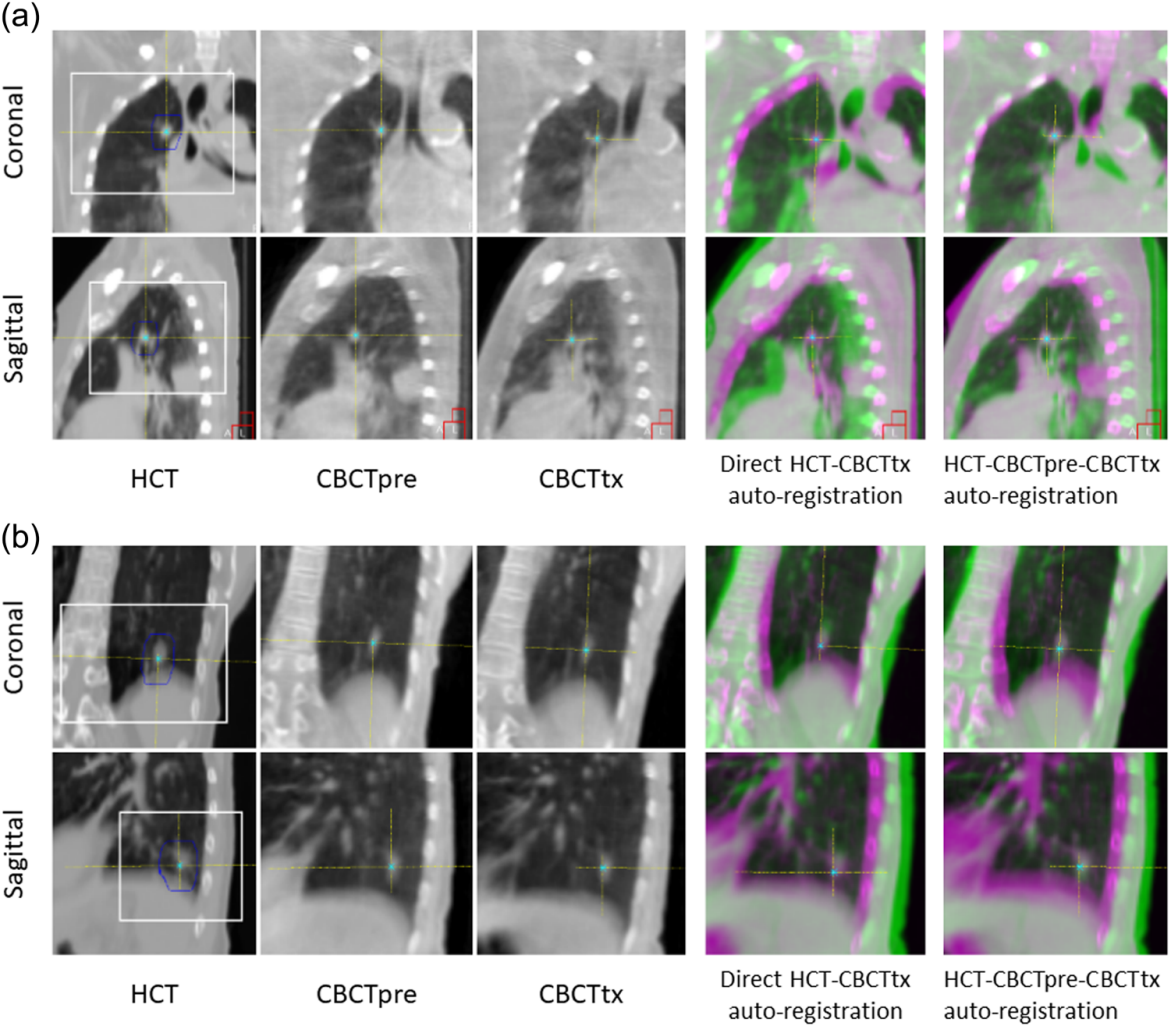
Images of the reference helical CT (HCT), cone beam computerized tomography (CBCT)pre, and CBCTtx for patient #29 (a) and patient #32 (b). The clip box (white) and mask (blue) regions of interest used during dual registration are displayed on the HCT images. The color blended images show the registration results between reference HCT (purple color) and CBCT (green color) from two auto‐registration methods

Figure 4a shows an example of a small lung nodule on HCT, CBCTpre, and CBCTtx, respectively (patient #29). The lesion appears to be well‐defined on the average HCT with relatively small motion amplitude. However, overall image contrast is degraded on CBCT due to increased noise, and tumor shrinkage is observed toward the last few fractions. The last two columns of color blending images show the mis‐matched and matched tumor between HCT and CBCTtx from two auto‐registration methods, respectively. On the matched images, the color complement at the center and the purple color residue on the edge reflect tumor's proper alignment and shrinkage, respectively. Figure 4b shows another example that presents large anatomical variation between HCT simulation and treatment (patient #32). In addition to the large motion amplitude, tumor baseline position variation is observed between HCT and CBCT as indicated by the increased distance from the diaphragm position. However, the averaged tumor shape and relative tumor to diaphragm distance appears to be consistent between CBCTs. The conventional direct HCT–CBCT auto‐registration produces an unsatisfactory result which is shown as the un‐complemented colors in the superior–inferior direction. Images in the last column demonstrate the successfully registered images from the proposed method where proper alignment is achieved for the tumor despite of the large variation of the diaphragm position.

## 4 DISCUSSIONS

We demonstrated the clinical feasibility of incorporating prior registration knowledge into the online image registration for lung SBRT. The accuracy of this method was found to be < 2 mm in each translational direction for the majority of cases. Larger uncertainties tended to occur along the superior–inferior direction which could be attributed to the facts that: (1) the tumor motion and clinician's manual adjustment often dominated in this direction; and (2) resolution of the HCT image was sparser in the SI direction than the other two directions (3.0 vs. 0.98 mm). The main advantage of the proposed method over conventional direct HCT–CBCT auto‐registration was that it demonstrated more robust performance when large differences existed between HCT and CBCT images. For the six cases that benefited most from the proposed method (#27–32), all of them had better image similarities between CBCTs than between HCT and CBCT. It was also this observation that initially prompted us to explore the proposed registration strategy. The causes of the large differences between HCT and CBCT seemed to be either related to the decreased image quality in CBCT or to the breathing and anatomical changes between HCT simulation and treatment delivery (Figure 4). Although the proposed method seems to be robust in these challenging scenarios, it is also important to point out here that large image differences between reference HCT and CBCT may indicate underlying tumor progression/regression or other internal anatomical changes that warrants a new simulation and treatment planning process.

The proposed method did not demonstrate significant difference in rotational parameters compared to the conventional method, which could be explained by two reasons. The first reason was that the rotational parameters were primarily determined from the clip‐box bone registration. Both HCT and CBCT images provided sufficient bone contrast, therefore bone registration results between two methods were expected to be similar. The second reason was related to the maximum 3° rotational range of the robotic six degrees‐of‐freedom couch (HexaPOD™, Elekta, Crawley, UK). During the manual adjustment, the clinician usually limited the rotational parameters to < ±3° and sometime tried to minimize them if possible due to the concerns that patients might involuntarily counteract large couch rotations. Nevertheless, as shown in Figure 3, the rotational errors from both methods were well < ±2° in the majority of patients. Previous studies showed that, for lung SBRT treatment, rotational errors up to 5° resulted in only minimal dosimetric changes for both target and organs at risk (OARs).^18^, ^19^ However, such a conclusion may vary depending on the proximity of an OAR to the target and the shape of the isodose distribution. In those situations, online dose re‐calculation may become necessary in order to evaluate the dosimetric effect and find the optimal correction strategy.

As discussed earlier, the proposed method may provide a more robust auto‐registration result for certain challenging registration cases. In clinical practices, these cases can be identified during the verification session or the earlier treatment session if the verification session is not routinely included in the workflow. There is ample time allowed to review, and peer‐review if necessary, the HCT–CBCTpre registration result. In our current study design, the CBCT from the verification session was chosen as the CBCTpre in order to create a more challenging testing scenario. Selecting the CBCTpre from the most recent treatment session is expected to provide better image similarities between two CBCTs, and thus may further improve the accuracy and robustness of the proposed method. At the time of this study, the whole process was not fully automated due to the lack of access to the source code of the XVI software. Manually generating the reference dataset and transferring image is just to utilize the current available XVI functions. However, these tedious manual processes could be done in an automatic fashion once fully integrated within XVI. It is anticipated that the proposed method has the potential to provide a more consistent auto‐registration result, speed up the online registration process, and improve patient comfort by reducing total on‐table time.

There are several limitations to the present study. First, the proposed method was only tested on one vendor's platform. The result may vary for other platforms that use different CBCT reconstruction and registration algorithms. The XVI grey value registration algorithm uses “correlation ratio” as the similarity measure which is different from the more commonly used “mutual information” measure for inter‐modality image registration.^11^, ^12^, ^13^, ^14^ The accuracy and robustness of the proposed method thus need to be fully validated when implemented on other image guidance platforms. Second, the conventional direct HCT–CBCT auto‐registration may not be fully optimized for each individual patient. Although previous study suggests that different clip‐box settings produce similar result for the dual registration method,^20^ the impact of mask setting on the registration result is still unclear. It is also necessary to note that we use the average HCT as the reference image and the mask registration is performed between the average HCT and the average CBCT. XVI provides the “Grey Value 4D (T)” registration option, where each phase CBCT image is registered independently with the mid‐phase HCT and the time‐averaged displacement was then calculated. The 4D mode may produce better result for tumor with large motion amplitude. However, there is still a lack of detailed assessment of the potential improvement from this new registration mode, and also a lack of consensus about which dataset is optimal as the reference image.^20^, ^21^, ^22^ The third limitation is the small number of test cases. However, considering the fact that the test patients were challenging cases selected from a relatively larger group, the advantage of addressing a challenging subset of patients is expected to hold. Another noteworthy limitation is related to the calculation of the rotational errors. Although the combined rotation angles were derived using matrix multiplications, which are not commutative, the residual rotational errors were simply calculated by subtracting each Euler angle from its corresponding ground‐truth. It is not uncommon for researchers to use such simple subtraction to calculate rotational errors when the rotational angles are small (cos(*t*) →1 and sin(*t*) →0).^23^, ^24^, ^25^

## 5 CONCLUSIONS

We tested the feasibility and accuracy of using previously registered CBCT image to assist online HCT–CBCT registration for lung SBRT. The proposed strategy is theoretically simple and does not require any processing of the raw data. Once fully integrated with the image registration platform, it has the potential to improve the online registration process by providing a reliable auto‐registration result.

## CONFLICT OF INTEREST

The authors declare that there is no conflict of interest that could be perceived as prejudicing the impartiality of the research reported.

## AUTHOR CONTRIBUTION

Jian Liang and Qiang Liu both designed the study, collected/analyzed the data, and prepared the paper. Inga S. Grills, Thomas Guerrero, and Craig Stevens provide patient data and performed clinical image registrations. Di Yan provided general supervision, made comments and feedback on the paper.

## Supporting information

Supporting information

## Data Availability

The data that support the findings of this study are available from the corresponding author upon reasonable request.
