# Supplementary material for: Using previously registered cone beam computerized tomography images to facilitate online computerized tomography to cone beam computerized tomography image registration in lung stereotactic body radiation therapy
Source: J Appl Clin Med Phys. 2022 Feb 2;23(4):e13549. doi: 10.1002/acm2.13549 (PMC8992944; doi:10.1002/acm2.13549)
Supplement: Supplementary file 1 — Supporting information [file ACM2-23-e13549-s001.docx]

**Using previously registered CBCT images to facilitate online CT to CBCT image registration in lung stereotactic body radiation therapy**

*Jian Liang, Qiang Liu, Inga S. Grills, Thomas Guerrero, Craig Stevens and Di Yan*

*Radiation Oncology, Beaumont Health System, Royal Oak, Michigan 48073, United States*

Running Title: Use prior knowledge for CBCT registration

Key words: Lung SBRT, CBCT, IGRT, Image registration

Author to whom correspondence should be addressed.

Qiang Liu

Radiation Oncology, Beaumont Health System, Royal Oak, Michigan 48073

Email: [qiang.liu@beaumont.org](mailto:qiang.liu@beaumont.org)

Tel: 248-551-7037

Author Contribution Statement: Jian Liang and Qiang Liu both designed the study, collected/analyzed the data, and prepared the manuscript. Inga S. Grills, Thomas Guerrero and Craig Stevens provide patient data and performed clinical image registrations. Di Yan provided general supervision, made comments and feedback on the manuscript.

Data Availability Statement: The data that support the findings of this study are available from the corresponding author upon reasonable request.

Conflict of interest or financial disclosures: None.
